# Supplementary material for: Descending networks transform command signals into population motor control
Source: Nature. 2024 Jun 5;630(8017):686–94. doi: 10.1038/s41586-024-07523-9 (PMC11186778; doi:10.1038/s41586-024-07523-9)
Supplement: Supplementary file 1 — This file contains Supplementary Discussion; Supplementary Tables 1–7; legends for Supplementary Tables 1–8 and Supplementary Videos; Supplementary References [file 41586_2024_7523_MOESM1_ESM.pdf]

nature portfolio

https://doi.org/10.1038/s41586-024-07523-9

Supplementary information

Descending networks transform command signals into population motor control

In the format provided by the authors and unedited

Supplementary Information
for Braun, Hurtak et al., *Nature* 2024

Descending networks transform command signals into population motor control

Jonas Braun*[1], Femke Hurtak*[1], Sibo Wang-Chen[1], and Pavan Ramdya[1†]

[1] Neuroengineering Laboratory, Brain Mind Institute & Interfaculty Institute of Bioengineering, EPFL, Lausanne, Switzerland

* equal contribution

† corresponding author: pavan.ramdya@epfl.ch

Table of Contents

Supplementary Information Tables 3
Supplementary Table 1 - Transgenic fly lines generated in this study............................ 3
Supplementary Table 2 - SpGAL4 fly lines used in this study 3
Supplementary Table 3 - List of antibodies used for tissue staining............................... 3
Supplementary Table 4 - Parameters used for behavior classification. 3
Supplementary Table 5 - Exact p-values for statistical tests in headless animal experiments 3
Supplementary Table 6 - Exact p-values for statistical tests in tibia-tarsus leg amputation animal experiments 3
Supplementary Table 7 - Acronyms for the brain neuropils 3
Supplementary Tables 4
Supplementary Table 8 - DN cluster analysis.. 4
Sheet 1: DN cluster behaviors. 4
Sheet 2: DN cluster VNC projections. 4
Sheet 3: Investigated DN clusters.. 4
Sheet 4: Equivalence DN names and root id. 4
Sheet 5: DN literature aggregation.. 4
Sheet 6: Connectivity statistics. 4
Supplementary Videos 5
Supplementary Video 1: DN-driven behavior and trial-averaged GNG-DN population activity.. 5
Supplementary Video 2: Comparing GNG-DN population activity for DN-driven versus natural behaviors. 5
Supplementary Video 3: DN-driven behavioral responses of animals that are intact, headless, or headless without ground contact. 5
Supplementary Video 4: Broadcaster DN-driven behavioral responses of animals that are intact, headless, or headless without ground contact. 5
Supplementary Video 5: Standalone DN-driven behavioral responses of animals that are intact, headless, or headless without ground contact. 5
Supplementary Video 6: DN-driven behavioral responses to optogenetic stimulation for intact and hindleg amputated animals. 5

Supplementary Information Tables

Available in the pdf file ‘Supplementary Information’.

Supplementary Table 1 - Transgenic fly lines generated in this study

Supplementary Table 2 - SpGAL4 fly lines used in this study

Supplementary Table 3 - List of antibodies used for tissue staining

Supplementary Table 4 - Parameters used for behavior classification.

Supplementary Table 5 - Exact p-values for statistical tests in headless animal experiments

Supplementary Table 6 - Exact p-values for statistical tests in tibia-tarsus leg amputation animal experiments

Supplementary Table 7 - Acronyms for the brain neuropils

Supplementary Tables

Available in the Excel spreadsheet 'Supplementary Table 8'.

Supplementary Table 8 - DN cluster analysis

Sheet 1: DN cluster behaviors.
A list showing which DNs are in which particular cluster.

Sheet 2: DN cluster VNC projections.
VNC projections for known DNs. Aside from MDN, these data were obtained from Cheong *et al.*, 2023.

Sheet 3: Investigated DN clusters.
A subset of sheet 1 showing the cluster associations for DNs investigated using optogenetics in this study: DNp09, aDN2, MDN, aDN1, DNa01, DNa02, DNb02, DNg14 and Mute.

Sheet 4: Equivalence DN names and root id.
List of DNs, providing the known names for the root ids used in FAFB.

Sheet 5: DN literature aggregation.
Reported behavioral phenotypes for DNs, including citations.

Sheet 6: Connectivity statistics.
Connectivity statistics in the DN-DN network for reported neurons.

Supplementary Videos

Supplementary Video 1: DN-driven behavior and trial-averaged GNG-DN population activity

For (bottom) behaviors driven by laser stimulation of (Part 1) DNp09, (Part 2) aDN2, (Part 3) MDN, (Part 4) control with no DN, (Part 5) DNp09 with T1 resected, shown are (top) stimulus-triggered averages of neural activity upon laser stimulation. Video shows $\Delta F/F$ processed GCaMP6s fluorescence. Red circle indicates laser stimulation. First four parts show flies from Figure 2b-d. For part 5, note that the most dorsal (top) part in the video of control fly 1 is just outside the cervical connective. Thus, observed changes over time are likely due to under-constrained motion correction outside of the cervical connective.

Supplementary Video 2: Comparing GNG-DN population activity for DN-driven versus natural behaviors.

For (bottom) behaviors in the same flies driven either (left) by laser stimulation of (Part 1) DNp09, (Part 2) aDN2, (Part 3) MDN, or (right) (Part 1) spontaneously, (Part 2) vapor-puff stimulation, (Part 3) spontaneously on a cylindrical treadmill, shown are (top) Stimulus-triggered averages of neural activity. Video shows $\Delta F/F$ processed GCaMP6s fluorescence. Red circle indicates laser stimulation. White circle indicates spontaneous/puff-driven behavior detection.

Supplementary Video 3: DN-driven behavioral responses of animals that are intact, headless, or headless without ground contact.

Responses to optogenetic stimulation of (Part 1) DNp09, (Part 2) aDN2, (Part 3) MDN for three flies (one animal per column). The same animal is studied intact on the spherical treadmill (top), headless on the spherical treadmill (middle), and headless while hanging without ground contact (bottom). Red circles indicate optogenetic laser stimulation.

Supplementary Video 4: Broadcaster DN-driven behavioral responses of animals that are intact, headless, or headless without ground contact.

Responses to optogenetic stimulation of (Part 1) DNb02, (Part 2) DNp42, (Part 3) DNa01, (Part 4) DNa02, (Part 5) aDN1 for three flies (one animal per column). Animals are studied intact on the spherical treadmill (top), headless on the spherical treadmill (middle), or headless while hanging without ground contact (bottom, except for DNp42). Red circles indicate optogenetic laser stimulation.

Supplementary Video 5: Standalone DN-driven behavioral responses of animals that are intact, headless, or headless without ground contact.

Responses to optogenetic stimulation of (Part 1) DNg14, (Part 2) oviDN, (Part 3) DNg11, (Part 4) mute for three flies (one animal per column). Animals are studied intact on the spherical treadmill (top), headless on the spherical treadmill (middle), or headless while hanging without ground contact (bottom). Red circles indicate optogenetic laser stimulation.

Supplementary Video 6: DN-driven behavioral responses to optogenetic stimulation for intact and hindleg amputated animals.

Behavioral responses of (top) intact, or (bottom) amputated animals during optogenetic stimulation of (Part 1) MDN with bilateral amputation of the hindlegs, (Part 2) DNp09 with bilateral amputation of the front legs, (Part 3) DNp09 with bilateral amputation of the midlegs, (Part 4) DNp09 with bilateral amputation of the front legs. Three flies are shown per

condition (columns). Amputations are at the tibia-tarsus joints. Red circles indicate optogenetic laser stimulation.

Supplementary Tables

ID	Chromosome X	Chromosome II	Chromosome III
1		20xUAS-CsChr.mVenus (attP40), 13xLexAop-opGCaMP6s (su(Hw)attP5)	DfdLexA / TM6B
2		20xUAS-CsChr.mVenus (attP40), 13xLexAop-opGCaMP6s (su(Hw)attP5)	13xLexAop-CD4-tdTomato (VK00033), DfdLexA / TM6B
3		LexOp-myr-TdTomato / CyO	DfdLexA / TM6B
4		LexOP-H2B::mCherry / CyO	DfdLexA / TM6B
5		LexAop-GtACR1 / CyO	DfdLexA / TM6B
6	20xUAS-CsChr.[mVenus]attP18	VT023490-p65.AD	VT44845.GAL4DBD
7	20xUAS-CsChr.[mVenus]attP18	R76F12-AD	R18C11-DBD
8	20xUAS-CsChr.[mVenus]attP18	VT023490-p65.AD	R38F04-GAL4.DBD

Supplementary Table 1: Transgenic fly lines generated in this study.

DN	Split name	Ref.	Source Stock	Stock n°	AD	DBD
MDN	SS03131	[1]	Janelia	n.a.	VT023490-p65.AD	VT44845.GAL4DBD
aDN2	n.a.	[2]	Julie Simpson	n.a.	R76F12-AD	R18C11-DBD
DNp09	SS01540	[3]	Bloomington	75903	VT023490-p65.AD	R38F04-GAL4.DBD
DNa01	SS00731	[4]	Bloomington	75862	R22C05-p65.AD	R56G08GAL4.DBD
DNa02	SS00730	[4]	Janelia	n.a.	75C10-p65ADZp	87D07-ZpGdbd
aDN1	n.a.	[2]	Julie Simpson	n.a.	18C11-p65AD	R71D01-DBD
Web	SS41434	[5]	Janelia	n.a.	VT023750-p65ADZp	VT043284-ZpGDBD
DNb02	SS01600	[4]	Bloomington	75901	R59B10-p65.AD	R21F05GAL4.DBD
Mute	SS42606	[5]	Janelia	n.a.	26C03-p65ADZp	84D10-ZpGDBD
DNp24	SS01046	[4]	Bloomington	75821	VT048835-p65.AD	R24C07GAL4.DBD
DNg30	SS02378	[4]	Bloomington	75872	R61A01-p65.AD	R13B05GAL4.DBD
DNg14	SS04158	[4]	Bloomington	75812	R53D12-p65.AD	VT018689GAL4.DBD
DNg11	SS01550	[4]	Bloomington	75949	VT037583-p65.AD	VT025598GAL4.DBD
oviDN	SS46540	[6]	Bloomington	86832	VT050660-p65.AD	VT028160-GAL4.DBD
DNb01	SS02383	[4]	Bloomington	75888	VT013121-p65.AD	R45H03-GAL4.DBD
DNg16	SS01543	[4]	Bloomington	75920	VT043288-p65.AD	VT028153GAL4.DBD
DNp42	SS57807	[7]	Bloomington	87282	R32A04-p65.AD	VT033636-GAL4.DBD

Supplementary Table 2: SpGAL4 fly lines used in this study.

figure	type	AB name	dilution	company	AB ID
a,b,c	1°	Anti-Bruchpilot (mouse) NC82	1:20	Dev. Studies Hybridoma Bank	NC82 1ml supernatant
a,c	1°	GFP Tag Rabbit	1:500	ThermoFisher	G10362
a,b,c	2°	Goat anti-Mouse Alexa 633	1:500	ThermoFisher	A21052
a,c	2°	Goat anti-Rabbit Alexa 488	1:500	ThermoFisher	A11008
b	1°	Living Colors DsRed	1:500	Takara	632496
b	1°	Chicken to GFP Anti-GFP	1:1000	abcam	ab13970
b	2°	Goat Anti-Rabbit (Cy3)	1:500	abcam	ab6939
b	2°	Goat Anti-Chicken (Alexa 488)	1:500	abcam	ab150169

Supplementary Table 3: List of antibodies used for immunofluorescence tissue staining in Extended Data Fig. 1.

Behavior	v_{abs} (mm/s)	v_{forw} (mm/s)	fr. leg height	ME front	ME mid	ME hind
Rest	≤ 0.75	n.a.	> 0	≤ 0.75	≤ 0.75	≤ 0.75
Forward walk	n.a.	> 1	n.a	n.a	n.a	n.a
Backward walk	n.a.	≤ -1	n.a	n.a	n.a	n.a
Anterior grooming 1	≤ 0.75	n.a.	≤ 0	n.a.	≤ 0.75	≤ 0.75
Anterior grooming 2	≤ 0.75	n.a.	> 0	> 0.75	≤ 0.75	≤ 0.75
Posterior grooming	≤ 0.75	n.a.	> 0	≤ 0.75	n.a.	> 0.75

Supplementary Table 4: Parameters used for behavior classification.

Figure	GAL4	Variable	Comparison	N	P-value
Figure 4b	DNp09	forw. vel.	intact/headless	5	0.006
Figure 4b	DNp09	forw. prob.	intact/headless	5	0.006
Figure 4c	aDN2	forw. vel.	intact/headless	5	0.018
Figure 4c	aDN2	groom prob.	intact/headless	5	0.006
Figure 4d	MDN	forw. vel.	intact/headless	5	0.500
Figure 4d	MDN	back prob.	intact/headless	5	0.265
Figure 4e	control	forw. vel.	intact/headless	5	0.500
Figure 4e	control	rest prob.	intact/headless	5	0.018
Figure 4f	DNp09 v. control	abd. contr.	headless/headless	5	0.006
Figure 4g	aDN2 v. control	fr. leg appr.	headless/headless	5	0.030
Figure 5f	DNb02	turn vel.	intact/headless	3/7	0.001
Figure 5f	control	turn vel.	intact/headless	5	0.047
Figure 5f	DNb02 v. control	turn vel.	headless/headless	7/5	0.313
Figure 5g	DNg14	abd. dip	intact/headless	9	0.144
Figure 5g	control	abd. dip	intact/headless	5	0.072
Figure 5g	DNg14 v. control	abd. dip	headless/headless	9/5	0.003
Extended Data Fig. 5a	DNp42	forw. vel	intact/headless	3	0.040
Extended Data Fig. 5a	control	forw. vel	intact/headless	3	0.040
Extended Data Fig. 5a	DNp42 v. control	forw. vel	headless/headless	3	0.191
Extended Data Fig. 5b	aDN1	fr. leg motion	intact/headless	5	0.002
Extended Data Fig. 5b	control	fr. leg motion	intact/headless	5	0.006
Extended Data Fig. 5b	aDN1 v. control	fr. leg motion	headless/headless	5	0.006
Extended Data Fig. 5b	aDN1	FeTi angle	intact/headless	5	0.008
Extended Data Fig. 5b	control	FeTi angle	intact/headless	5	0.417
Extended Data Fig. 5b	aDN1 v. control	FeTi angle	headless/headless	5	0.148
Extended Data Fig. 5b	aDN1	fr. leg appr.	intact/headless	5	0.149
Extended Data Fig. 5b	control	fr. leg appr.	intact/headless	5	0.072
Extended Data Fig. 5b	aDN1 v. control	fr. leg appr.	headless/headless	5	0.030
Extended Data Fig. 5c	DNa01	turn vel.	intact/headless	6	0.003
Extended Data Fig. 5c	control	turn vel.	intact/headless	5	0.047
Extended Data Fig. 5c	DNa01 v. control	turn vel.	headless/headless	6/5	0.206
Extended Data Fig. 5c	DNa01	side vel.	intact/headless	6	0.003
Extended Data Fig. 5c	control	side vel.	intact/headless	5	0.047
Extended Data Fig. 5c	DNa01 v. control	side vel.	headless/headless	6/5	0.392
Extended Data Fig. 5d	DNb02	turn vel.	intact/headless	3/7	0.001
Extended Data Fig. 5d	control	turn vel.	intact/headless	5	0.047
Extended Data Fig. 5d	DNb02 v. control	turn vel.	headless/headless	7/5	0.313
Extended Data Fig. 5d	DNb02	forw. vel.	intact/headless	3/7	0.028
Extended Data Fig. 5d	control	forw. vel.	intact/headless	5	0.500
Extended Data Fig. 5d	DNb02 v. control	forw. vel.	headless/headless	7/5	0.313
Extended Data Fig. 5e	DNa02	turn vel.	intact/headless	6	0.002
Extended Data Fig. 5e	control	turn vel.	intact/headless	5	0.047
Extended Data Fig. 5e	DNa02 v. control	turn vel.	headless/headless	6/5	0.158
Extended Data Fig. 5e	DNa02	side vel.	intact/headless	6	0.003
Extended Data Fig. 5e	control	side vel.	intact/headless	5	0.047
Extended Data Fig. 5e	DNa02 v. control	side vel.	headless/headless	6/5	0.085
Extended Data Fig. 6a	oviDN	abd. dip	intact/headless	3	0.331
Extended Data Fig. 6a	control	abd. dip	intact/headless	3	0.191
Extended Data Fig. 6a	oviDN v. control	abd. dip	headless/headless	3	0.040
Extended Data Fig. 6b	DNg11	TiTa angle	intact/headless	6	0.468
Extended Data Fig. 6b	control	TiTa angle	intact/headless	3	0.040
Extended Data Fig. 6b	DNg11 v. control	TiTa angle	headless/headless	6/3	0.014
Extended Data Fig. 6c	Mute	ovi. ext.	intact/headless	3	0.500
Extended Data Fig. 6c	control	ovi. ext.	intact/headless	5	0.338
Extended Data Fig. 6c	Mute v. control	ovi. ext.	headless/headless	3/5	0.117
Extended Data Fig. 6d	DNg14	abd. dip	intact/headless	9	0.144
Extended Data Fig. 6d	control	abd. dip	intact/headless	5	0.072
Extended Data Fig. 6d	DNg14 v. control	abd. dip	headless/headless	9/5	0.003

Supplementary Table 5: Exact p-values for statistical tests in headless animal experiments.

GAL4	Legs amputated	Comparison	N	P-value
MDN	hind	intact/amputated	4/5	0.010
control	hind	intact/amputated	10/3	0.466
MDN v. control	hind	amputated/amputated	4/5	0.068
DNp09	hind	intact/amputated	3/3	0.191
control	hind	intact/amputated	10/3	0.466
DNp09 v. control	hind	amputated/amputated	3/3	0.040
DNp09	middle	intact/amputated	3/4	0.026
control	middle	intact/amputated	10/3	0.336
DNp09 v. control	middle	amputated/amputated	3/4	0.026
DNp09	front	intact/amputated	3/4	0.026
control	front	intact/amputated	10/3	0.075
DNp09 v. control	front	amputated/amputated	3/4	0.026

Supplementary Table 6: Exact p-values for statistical tests in tibia-tarsus leg amputation animal experiments (**Extended Data Fig. 10**).

Acronym	Neuropil	Region
AL	antennal lobe	Antennal Lobe
AME	accessory medulla	Optic Lobe
AMMC	antennal mechanosensory and motor center	Periesophageal Neuropils
AOTU	anterior optic tubercle	Ventrolateral Neuropils
ATL	antler	Inferior Neuropils
AVLP	anterior VLP (ventrolateral protocerebrum)	Ventrolateral Neuropils
BU	bulb	Lateral Complex
CAN	cantle	Periesophageal Neuropils
CRE	crepine	Inferior Neuropils
EPA	epaulette	Ventromedial Neuropils
FB	fanshaped body	Central Complex
FLA	flange	Periesophageal Neuropils
GA	gall	Lateral Complex
GNG	gnathal ganglia	Gnathal Ganglia
GOR	gorget	Ventromedial Neuropils
IB	inferior bridge	Inferior Neuropils
ICL	inferior clamp	Inferior Neuropils
IPS	inferior posterior slope	Ventromedial Neuropils
LAL	lateral accessory lobe	Lateral Complex
LH	lateral horn	Lateral Horn
LOP	lobula plate	Optic Lobe
LO	lobula	Optic Lobe
MB_CA	pedunculus	Mushroom Body
MB_ML	vertical lobe	Mushroom Body
MB_PED	medial lobe	Mushroom Body
MB_VL	calyx	Mushroom Body
NO	noduli	Central Complex
OCG	ocellar ganglion	Ocelli
PB	fanshaped body	Central Complex
PLP	posteriorlateral protocerebrum	Ventrolateral Neuropils
PRW	prow	Periesophageal Neuropils
PVLP	posterior VLP (ventrolateral protocerebrum)	Ventrolateral Neuropils
SAD	saddle	Periesophageal Neuropils
SCL	superior clamp	Inferior Neuropils
SIP	superior intermediate protocerebrum	Superior Neuropils
SLP	superior lateral protocerebrum	Superior Neuropils
SMP	superior medial protocerebrum	Superior Neuropils
SPS	superior posterior slope	Ventromedial Neuropils
UNASGD	unassigned	
VES	vest	Ventromedial Neuropils
WED	wedge	Ventrolateral Neuropils

Supplementary Table 7: Acronyms for the brain neuropils used in **Extended Data Fig. 7** based on ref. [8]

Supplementary Discussion

Alternative mechanisms for DN recruitment In this work we highlight that direct synaptic connections between DNs are sufficient to explain the recruitment of DN populations by command-like DNs. However, there are other mechanisms may contribute in parallel.

The first additional mechanism is via ascending feedback to the brain. Interestingly, the GNG receives a large number of inputs from ascending neurons (ANs) that project from the VNC to the brain [9,10]. Among these are a set of ANs which play a role in the decision between locomotion and feeding [11]. Connections from ANs may thus allow DNs to integrate information from the VNC to regulate switching between actions. DN-DN recruitment might also potentially arise indirectly via a DN-AN-DN 'zigzag' motif that has previously been observed in low numbers in the brain connectome of *Drosophila* larvae [12] (i.e., a DN targets an AN in the VNC which then projects back to the brain targeting a different DN). In the adult, ANs encompass 17% of all DN post-synaptic partners in the VNC [13] and 10% of all DN pre-synaptic partners in the brain. Although our experiments in headless animals cannot test the contribution of ANs (decapitation eliminates both DN-DN and putative DN-AN-DN connections), our VNC resection experiments show that ANs are not required for DN recruitment. These connections are far less numerous than direct DN-DN connectivity in the larval brain. Efforts aiming to bridge the existing brain and VNC connectomes [8,14,15] and to generate complete adult nervous system connectomes will further reveal the relative contribution of ANs to DN recruitment.

DN recruitment might also arise even more indirectly via sensory feedback during a change in behavioral state: Active DNs may drive a new behavior, resulting in limb sensory feedback that in turn may be transmitted via ANs to influence other DNs. We would expect to see such sensory-induced DN activation in spontaneous behavior but we instead observe that, in general, fewer DNs are strongly activate during spontaneous behavior compared with during optogenetically elicited behavior. This argues that strong GNG-DN activation is a specific response to optogenetic stimulation of command-like DNs. In particular, we found that the 10 most active neurons during DNp09 stimulation are not active during spontaneous forward walking. These results, as well as those from our VNC resection experiments blocking ascending feedback to the brain, suggest that DN recruitment likely does not result from sensory feedback arising during optogenetically-induced changes in behavioral state.

We find that DNp09 stimulation elicits additional DN activity beyond what is normally seen during spontaneous walking. This suggests a distinction between DN populations becoming active during spontaneously generated, sensory-induced, and optogenetically activated walking. We note that we previously observed that a large fraction of DNs in the CRG (rather than GNG-DNs recorded in this study) become active during spontaneous and odor-evoked forward walking [16]. Thus, spontaneously generated and sensory-induced walking may principally be driven by CRG-DNs. For example, DNp09 are thought to mediate courtship-related forward walking [3]. The possibility that DNp09 is active only during specific courtship-related behavioral contexts and inactive during spontaneous walking is supported by recent electrophysiological evidence [17]. Thus, it appears that forward walking can be controlled by distinct DNs depending on the context.

The various roles of DNs in motor control In our work we hypothesized that DNs might each drive 'motor primitives' which, when composed, give rise to complete, coordinated behaviors. Beyond a direct role in motor control other DNs may be modulatory, controlling behavioral vigor or persistence. Other DNs may be inhibitory, potentially playing an important role in action selection. Finally, other DNs may have a role in 'gating' behaviors by increasing the excitability of downstream motor circuits.

For DNs driving specific motor actions, we propose a framework in which DNs coordinate complex behaviors by recruiting additional DNs driving simpler motor primitives. Further evidence for such a model could come from activating command-like DNs while silencing downstream DNs. For example, DNp09 is connected to and requires the actions of a large number of DNs to drive movements of the six legs for goal-directed walking during courtship [3]. Both DNp09 (for forward walking) and MDN (for backward walking) synapse upon DNa01 and DNa02, two DNs involved in turning [18,19]. Thus, one might silence DNa01 or DNa02 to test the prediction that they control specific turning kinematics during either asymmetric forward or backward walking. Notably, we found that DNa01 and DNa02 also synapse onto other DNs. However, their activation alone (i.e., in headless animals) is not sufficient to drive turning. Thus, DNa01 and DNa02 may also sit atop the hierarchy of DN

recruitment, recruiting other DNs which control individual leg degrees of freedom. As an example of a DN controlling similarly few degrees of freedom, we found that stimulating DNg14 drives lowering of the abdomen. In line with it being at lowest level in a DN hierarchy, DNg14 does not have any downstream DNs, but receive inputs from twelve upstream DNs.

References

[1] Bidaye, S. S., Machacek, C., Wu, Y. & Dickson, B. J.. Neuronal Control of Drosophila Walking Direction. *Science* **344**, 97–101 (2014).

[2] Hampel, S., Franconville, R., Simpson, J. H. & Seeds, A. M.. A neural command circuit for grooming movement control. *eLife* **4**, e08758 (2015).

[3] Bidaye, S. S. *et al.* Two Brain Pathways Initiate Distinct Forward Walking Programs in Drosophila. *Neuron* **108**, 469-485 (2020).

[4] Namiki, S. *et al.* The functional organization of descending sensory-motor pathways in Drosophila. *eLife* **7**, e34272 (2018).

[5] Sterne, G. R., Otsuna, H., Dickson, B. J. & Scott, K.. Classification and genetic targeting of cell types in the primary taste and premotor center of the adult Drosophila brain. *eLife* **10**, e71679 (2021).

[6] Wang, F. *et al.* Neural circuitry linking mating and egg laying in Drosophila females. *Nature* **579**, 101–105 (2020).

[7] Huoviala, P. *et al.* Neural circuit basis of aversive odour processing in Drosophila from sensory input to descending output. *bioRxiv* 394403 (2020).

[8] Dorkenwald, S. *et al.* Neuronal wiring diagram of an adult brain. *bioRxiv* 2023.06.27.546656 (2023).

[9] Chen, C. *et al.* Ascending neurons convey behavioral state to integrative sensory and action selection brain regions. *Nature Neuroscience* **26**, 682–695 (2023).

[10] Tsubouchi, A. *et al.* Topological and modality-specific representation of somatosensory information in the fly brain. *Science* **358**, 615–623 (2017).

[11] Mann, K., Gordon, M. D. & Scott, K.. A Pair of Interneurons Influences the Choice between Feeding and Locomotion in Drosophila. *Neuron* **79**, 754–765 (2013).

[12] Winding, M. *et al.* The connectome of an insect brain. *Science* **379**, eadd9330 (2023).

[13] Cheong, H. S. J. *et al.* Transforming descending input into behavior: The organization of premotor circuits in the Drosophila Male Adult Nerve Cord connectome. *bioRxiv* 2023.06.07.543976 (2023).

[14] Phelps, J. S. *et al.* Reconstruction of motor control circuits in adult Drosophila using automated transmission electron microscopy. *Cell* **184**, 759–774 (2021).

[15] Marin, E. C. *et al.* Systematic annotation of a complete adult male Drosophila nerve cord connectome reveals principles of functional organisation. *bioRxiv* 2023.06.05.543407 (2023).

[16] Aymanns, F., Chen, C. & Ramdya, P.. Descending neuron population dynamics during odor-evoked and spontaneous limb-dependent behaviors. *eLife* **11**, e81527 (2022).

[17] Yang, H. H. *et al.* Fine-grained descending control of steering in walking Drosophila. *bioRxiv* 2023.10.15.562426 (2023).

[18] Chen, C. *et al.* Imaging neural activity in the ventral nerve cord of behaving adult Drosophila. *Nature Communications* **9**, 4390 (2018).

[19] Rayshubskiy, A. *et al.* Neural control of steering in walking Drosophila. *bioRxiv* 2020.04.04.024703 (2020).
