# Supplementary material for: Descending networks transform command signals into population motor control
Source: Nature. 2024 Jun 5;630(8017):686–94. doi: 10.1038/s41586-024-07523-9 (PMC11186778; doi:10.1038/s41586-024-07523-9)
Supplement: Supplementary file 2 — Reporting Summary [file 41586_2024_7523_MOESM2_ESM.pdf]

nature portfolio

Corresponding author(s): Pavan Ramdya

Last updated by author(s): Apr 15, 2024

Reporting Summary

Nature Portfolio wishes to improve the reproducibility of the work that we publish. This form provides structure for consistency and transparency in reporting. For further information on Nature Portfolio policies, see our Editorial Policies and the Editorial Policy Checklist.

Statistics

For all statistical analyses, confirm that the following items are present in the figure legend, table legend, main text, or Methods section.

n/a	Confirmed	
☐	☒	The exact sample size (*n*) for each experimental group/condition, given as a discrete number and unit of measurement
☐	☒	A statement on whether measurements were taken from distinct samples or whether the same sample was measured repeatedly
☐	☒	The statistical test(s) used AND whether they are one- or two-sided<br>*Only common tests should be described solely by name; describe more complex techniques in the Methods section.*
☐	☒	A description of all covariates tested
☐	☒	A description of any assumptions or corrections, such as tests of normality and adjustment for multiple comparisons
☐	☒	A full description of the statistical parameters including central tendency (e.g. means) or other basic estimates (e.g. regression coefficient) AND variation (e.g. standard deviation) or associated estimates of uncertainty (e.g. confidence intervals)
☐	☒	For null hypothesis testing, the test statistic (e.g. *F*, *t*, *r*) with confidence intervals, effect sizes, degrees of freedom and *P* value noted<br>*Give P values as exact values whenever suitable.*
☒	☐	For Bayesian analysis, information on the choice of priors and Markov chain Monte Carlo settings
☒	☐	For hierarchical and complex designs, identification of the appropriate level for tests and full reporting of outcomes
☐	☒	Estimates of effect sizes (e.g. Cohen's *d*, Pearson's *r*), indicating how they were calculated

Our web collection on statistics for biologists contains articles on many of the points above.

Software and code

Policy information about availability of computer code

Data collection	Two-photon microscope images were acquired using ThorImage 3.2 software. Data synchronization was performed using ThorSync 3.2 software. Behavior images were acquired using custom Python scripts. Optogenetic stimulation was controlled using a custom Arduino 1.8.13 script.
Data analysis	Data analyses were performed using custom code written in Python 3. The code is available in the following repository: https://github.com/NeLy-EPFL/dn_networks<br>Fiji v.2.9.0 software was used to sum z-projections of image z-stacks and to combine monochromatic images into RGB images.<br>SLEAP v1.3.0 was used to perform 2D pose estimation.

For manuscripts utilizing custom algorithms or software that are central to the research but not yet described in published literature, software must be made available to editors and reviewers. We strongly encourage code deposition in a community repository (e.g. GitHub). See the Nature Portfolio guidelines for submitting code & software for further information.

Data

Policy information about availability of data

All manuscripts must include a data availability statement. This statement should provide the following information, where applicable:

- Accession codes, unique identifiers, or web links for publicly available datasets
- A description of any restrictions on data availability
- For clinical datasets or third party data, please ensure that the statement adheres to our policy

Data are available at:
https://dataverse.harvard.edu/dataverse/dn_networks
DOI are:
https://doi.org/10.7910/DVN/6IL0X3
https://doi.org/10.7910/DVN/K0WMM4
https://doi.org/10.7910/DVN/TZK8FA
https://doi.org/10.7910/DVN/INYAYV
https://doi.org/10.7910/DVN/HNGVGA
These repositories include processed data required to reproduce the figures for each fly. Due to data storage limits, these do not include raw behavior camera images or raw two-photon imaging files which are available upon reasonable request. This repository includes: all behavioral and neural time series required to reproduce Figures describing experimental data; Acquisition Metadata files; Confocal images; SLEAP pose estimation model.
The female adult fly brain (FAFB) connectomics dataset from Codex (version hosted on Codex as of August 3, 2023, FlyWire materialization snapshot 630) can be found at:
https://codex.flywire.ai/api/download.

Research involving human participants, their data, or biological material

Policy information about studies with human participants or human data. See also policy information about sex, gender (identity/presentation), and sexual orientation and race, ethnicity and racism.

Reporting on sex and gender	N/A
Reporting on race, ethnicity, or other socially relevant groupings	N/A
Population characteristics	N/A
Recruitment	N/A
Ethics oversight	N/A

Note that full information on the approval of the study protocol must also be provided in the manuscript.

Field-specific reporting

Please select the one below that is the best fit for your research. If you are not sure, read the appropriate sections before making your selection.

☒ Life sciences ☐ Behavioural & social sciences ☐ Ecological, evolutionary & environmental sciences

For a reference copy of the document with all sections, see nature.com/documents/nr-reporting-summary-flat.pdf

Life sciences study design

All studies must disclose on these points even when the disclosure is negative.

Sample size

Sample sizes were chosen based on convention in the field based on expected inter-animal variability from published results and pilot experiments. In total, we performed experiments with 176 flies:
(1) 20 flies for two-photon recordings during optogenetic stimulation after the fly was walking Fig 2). We have at least 3 samples per genotype.
(2) 25 flies to fine-tune and characterize the optogenetic stimulation system (Supp Fig 1; 9 overlap with (1)). We have at least 3 samples per genotype.
(3) 5 flies to examine spontaneous behavioral responses (Supp. Fig 2., 3 overlap with (1))
(4) 6 Flies to test DN recruitment upon VNC resection (Supp. Fig. 3). We have 3 samples per genotype.
(5) 20 flies for headless experiments (Fig. 4). We have at least 5 samples per genotype.
(6) 69 flies to test model predictions (Fig. 5 and Supp. Figs. 6,7). We have at least 3 samples per genotype.
(7) 35 flies to test forward and backward walking with amputated tarsi (Supp. Fig. 4). We have at least 3 samples per genotype.
(6) 5 flies for confocal imaging (Supp. Fig. 1)
(7) 11 flies for two-photon recordings during optogenetic responses after the fly was resting (Supp. File 1; 7 overlap with (1))

Data exclusions	Data from two-photon recordings of behaving flies were excluded for animals and trials in which we observed abnormal limb movements, or low vitality. Two-photon imaging data were also excluded if they suffered from optical occlusions due to tissue debris, or extreme motion artifacts resulting from animal behavior. Both of these exclusions were performed based on a quantitative scoring system. In experiments with headless flies, we only retained data from flies that were not visibly injured after decapitation (i.e., all limbs were still able to move).
Replication	For the two-photon recordings in Figure 2, 3-9 replicates were recorded for each genotype.<br>For the headless experiments shown in figure 4, five replicates were recorded for each genotype. For the experiments testing model predictions in Figure 5 and Supp. Figure 3, 3-9 replicates were recorded for each genotype.<br>For other control experiments (Supp. Fig 1d-g, Supp Fig 3, Supp Fig 4,) at least 3 replicates were recorded for each genotype.
Randomization	Experiments were not randomized because of the automated nature of the data analysis.
Blinding	Experimenters were not blinded in this study due to obvious behavioral phenotypes for specific genotypes during optogenetic activation. Additionally, for specific experiments, a behavioral phenotype was required to establish the health of the animals in question.

Reporting for specific materials, systems and methods

We require information from authors about some types of materials, experimental systems and methods used in many studies. Here, indicate whether each material, system or method listed is relevant to your study. If you are not sure if a list item applies to your research, read the appropriate section before selecting a response.

Materials & experimental systems

n/a	Involved in the study
☐	☒ Antibodies
☒	☐ Eukaryotic cell lines
☒	☐ Palaeontology and archaeology
☐	☒ Animals and other organisms
☒	☐ Clinical data
☒	☐ Dual use research of concern
☒	☐ Plants

Methods

n/a	Involved in the study
☒	☐ ChIP-seq
☒	☐ Flow cytometry
☒	☐ MRI-based neuroimaging

Antibodies

Antibodies used	Anti-Bruchpilot (mouse) NC82, Dev. Studies Hybridoma Bank (DSHB), NC82 1ml supernatant<br>GFP Tag Rabbit, ThermoFisher, G10362<br>Goat anti-Mouse Alexa 633, ThermoFisher, A21052<br>Goat anti-Rabbit Alexa 488, ThermoFisher, A11008<br>Living Colors DsRed, Takara, 632496<br>Chicken to GFP Anti-GFP, abcam, ab13970<br>Goat Anti-Rabbit (Cy3), abcam, ab6939<br>Goat Anti-Chicken (Alexa 488), abcam, ab150169
Validation	Primary antibodies were validated by the suppliers as follows:<br>GFP Tag Rabbit (G10362) was verified by Relative expression<br>Living Colors DsRed, (632496) was validated by western blot.<br>Chicken to GFP (ab13970) was validated by western blot.<br>No manufacturer notes are available for the validation of other primary antibodies. No additional validation was performed.<br>- DSHB - https://dshb.biology.uiowa.edu/nc82<br>- Thermofisher - https://www.thermofisher.com/antibody/product/<br>- Takara Biomedical Technology - https://www.takarabio.com/products/antibodies-and-elisa/fluorescent-protein-antibodies/red-fluorescent-protein-antibodies<br>- https://www.abcam.com/products

Animals and other research organisms

Policy information about studies involving animals; ARRIVE guidelines recommended for reporting animal research, and Sex and Gender in Research

Laboratory animals	Female Drosophila melanogaster flies 3-8 days post-eclosion (dpe) from the following driver lines were used in this study:<br>Split GAL4 lines (targeting DNp09, aDN2, MDN, aDN1, DNa01, DNa02, DNb02, DNg14, Mute, Web, DNp24, DNg30, DNg11, oviDN, DNb01, DNg16, DNp42).<br>20xUAS-CsChr.mVenus (attP40), 13xLexAop-opGCaMP6s (su(Hw)attP5); DfdLexA / TM6B<br>20xUAS-CsChr.mVenus (attP40), 13xLexAop-opGCaMP6s (su(Hw)attP5); 13xLexAop-CD4-tdTomato (VK00033), DfdLexA / TM6B

	LexOp-myr-TdTomato / CyO; DfdLexA / TM6B<br>LexOP-H2B::mCherry / CyO; DfdLexA / TM6B<br>LexAop-GtACR1 / CyO; DfdLexA / TM6<br>20xUAS-CsChr.[mVenus]attP18; spGAL4-AD; spGAL4-DBD (see Methods)<br>PR (Phinney Ridge wild type) flies, Canton-S wild type flies
Wild animals	No wild animals were used.
Reporting on sex	All studies were performed on female flies due to their larger body size. This property facilitates neural data analysis and behavioral quantification.
Field-collected samples	No field-collected samples were used
Ethics oversight	All experiments were performed in compliance with relevant national (Switzerland) and institutional (EPFL) ethical regulations

Note that full information on the approval of the study protocol must also be provided in the manuscript.
